# Germline *MC1R* Variant Status and Efficacy of Immune Checkpoint Inhibitors in Patients With Advanced Melanoma

**DOI:** 10.1111/pcmr.70050

**Published:** 2025-09-09

**Authors:** Muyi Yang, Suzanne Egyhazi Brage, Jan Lapins, Vitali Grozman, Fernanda Costa Svedman, Veronica Höiom, Hildur Helgadottir

**Affiliations:** ^1^ Department of Oncology and Pathology Karolinska Institutet Stockholm Sweden; ^2^ Dermatology and Venereology Unit, Department of Medicine Huddinge Karolinska Institutet Stockholm Sweden; ^3^ Department of Dermatology Karolinska University Hospital Stockholm Sweden; ^4^ Section of Thoracic Radiology, Department of Imaging and Physiology Karolinska University Hospital Stockholm Sweden; ^5^ Department of Molecular Medicine and Surgery Karolinska Institutet Stockholm Sweden; ^6^ Theme Cancer Karolinska University Hospital Stockholm Sweden

**Keywords:** germline genetics, immune checkpoint inhibitors, *MC1R*, melanoma, PD‐1 inhibitors

## Abstract

The melanocortin‐1‐receptor (MC1R*)* has a key role in melanocyte pigmentation regulation. Certain *MC1R* germline genetic variants (*R* alleles) result in deficient melanin production and are associated with red hair, freckling, UV sensitivity, and melanoma susceptibility. We aimed to address whether inherited polymorphisms in *MC1R* impact the efficacy of immune checkpoint inhibitors (ICI) in patients with metastatic melanoma. Patients with advanced melanoma undergoing ICI treatment were genotyped for *MC1R* variants. The patients were grouped by their germline *MC1R R* variants (≥ 1 or 0) and followed for treatment response, progression‐free survival (PFS) and overall survival (OS). Of the 103 patients included, 39 (37.9%) had at least one *MC1R R* allele (*MC1R*‐R‐carriers), whereas 64 patients did not harbor any *R* allele (*MC1R*‐R‐non‐carriers). The hazard ratio (HR) for PFS in *MC1R*‐R‐carriers was 0.60, (95% CI 0.37–0.98, *p* = 0.043). The HR for OS was 0.63 (95% CI 0.37–1.08, *p* = 0.091). While *MC1R* is closely associated with melanoma susceptibility, its impact on ICI efficacy has not been explored previously. *MC1R*‐R‐carriers with metastatic melanoma had improved PFS when treated with ICIs. If validated in larger cohorts, *MC1R* genotyping may serve as a factor helping to predict response to ICIs in melanoma patients.

Inherited genetic variability in the melanocortin 1 receptor (*MC1R*) gene is a well‐known determinant for the risk of cutaneous melanoma (Hoiom et al. 2009; Stefanaki et al. 2024). *MC1R* encodes a transmembrane G protein‐coupled receptor expressed on the surface of melanocytes and is one of the key genes involved in the regulation of pigmentation (Guida et al. 2022). Certain missense variants in *MC1R* reduce the ability of melanocytes to produce the dark, protective pigment eumelanin. The variants associated with the most deficient production have been designated *R* alleles and are more common in individuals of North European descent. In a study in the Swedish population, 33% of the healthy Swedish population had at least one *MC1R R* allele, whereas the corresponding number for patients diagnosed with primary cutaneous melanoma was 54% (Hoiom et al. 2009). While *MC1R* has been implicated as a major factor for melanoma susceptibility, its impact on survival from metastatic melanoma and treatment efficacy remains unexplored.

The aim of our study was to evaluate if inherited *MC1R R* alleles are predictive of the efficacy of immune checkpoint inhibitors (ICIs) in melanoma patients. Blood samples were collected from patients with unresectable metastatic cutaneous melanoma, treated with ICI (anti‐CTLA‐4 and/or anti‐PD‐1) at the Department of Oncology, Karolinska University Hospital, Stockholm, Sweden in the years 2012–2021. A detailed description of patient accrual, *MC1R* testing, follow‐up, and statistical analyses is available in the Supporting Information. Informed consent was attained from all patients, and the study was approved by the Swedish Ethical Review Authority. In total, 103 patients with metastatic melanoma were included; 39 (37.9%) were *MC1R*‐R‐carriers, and 64 (62.1%) were *MC1R*‐R‐non‐carriers (Table S1). A detailed report on the patient characteristics, identified *MC1R* variants, and treatment efficacy is available in the Supporting Information. Studies conducted before the introduction of ICIs in melanoma treatment have demonstrated that patients diagnosed with a primary melanoma with inherited *MC1R* variants have improved melanoma‐specific survival compared to patients with consensus *MC1R* alleles (Davies et al. 2012; Taylor et al. 2015). Interestingly, the frequency of *MC1R* R alleles in this cohort of Swedish patients with metastatic melanoma was lower than has been demonstrated for Swedish patients diagnosed with primary cutaneous melanoma (38% vs. 54%) (Hoiom et al. 2009). This difference could result from the seemingly less aggressive nature of cutaneous melanomas in *MC1R*‐R‐carriers, leading to a lower portion of such carriers among patients with metastatic disease.

As expected, the *MC1R*‐R‐carriers in the study had, compared to the *MC1R*‐R‐non‐carriers, a significantly higher frequency of Fitzpatrick phototype I‐II (68.8% vs. 42.6%, *p* = 0.020) and red hair (20.0% vs. 4.5%, *p* = 0.044); see Table S1. Primary melanomas of superficial spreading (SSM) type were also significantly more frequent (61.5% vs. 27.8%), while the other histological subtypes were of lower frequency in the *MC1R*‐R‐carriers compared to non‐carriers (*p* = 0.022). Further, metastatic melanoma of unknown primary was significantly less frequent among the *MC1R*‐R‐carriers compared to non‐carriers (10.3% vs. 26.6%, *p* = 0.030). The majority were treated with PD‐1 inhibitor monotherapy (86.4%), and a smaller proportion with CTLA‐4 inhibitor monotherapy (1.9%) or CTLA‐4/PD‐1 inhibitor combination therapy (11.7%). There were no significant differences in the carriers compared to non‐carriers for factors known to influence ICI treatment outcome, including sex, age, tumor stage, LDH, or previous treatments or regimen received. In a univariate analysis, having had an SSM or a nodular (NM) primary melanoma was significantly associated with an improved PFS (HR 0.48, 95% CI 0.24–0.98), while other primary tumor or patient characteristics (including pigmentation phenotype) were not associated with any significant differences in PFS or OS (Table S2). However, in the metastatic setting, tumor stage, LDH, and previous treatments were all significantly associated with both PFS and OS. The radiological response rate (complete or partial response) and disease control rate (complete or partial response) or stable disease were 53.8% versus 50.0% and 74.4% versus 59.4% in the *MC1R*‐R‐carriers and non‐carriers, *p* = 0.702 and *p* = 0.056, respectively (Table S3). The median progression‐free survival (PFS) was 18 months (95% CI 15–21 months) in *MC1R*‐R‐carriers vs. 11 months (95% CI 8–13) in non‐carriers, *p* = 0.033 (Figure 1). The overall survival (OS) was 38 months (95% CI 25–56) in *MC1R*‐R‐carriers vs. 21 months (95% CI 18–27) in non‐carriers, *p* = 0.080. In a univariate analysis, HR for PFS was 0.60 (95% CI 0.37–0.98, *p* = 0.043), and HR for OS was 0.63 (95% CI 0.37–1.08, *p* = 0.091). When adjusting for different patient, tumor, and treatment‐related factors, HR for PFS and OS remained < 0.75 in favor of the *MC1R*‐R‐carriers, except when adjusting for factors associated with the primary melanoma; the survival advantage did not persist (Table 1). This indicates that the primary melanoma histological subtype, at least in part, is an intermediary factor associated with a survival advantage in ICI‐treated *MC1R*‐R‐carriers. In a previous study, a poorer efficacy of BRAF inhibitors was observed in metastatic melanoma patients with germline *MC1R* variants (R), possibly related to altered p38 MAP kinase phosphorylation (Guida et al. 2016). In our cohort, the efficacy of BRAF inhibitors was not specifically assessed, but as differing BRAF inhibitor efficacy in *MC1R‐R*‐carriers and non‐carriers could potentially affect the survival outcome, comparisons were done on the BRAF mutation status and BRAF inhibitor treatment. No significant differences were, however, noted between *MC1R‐R*‐carriers and non‐carriers in the frequency of the tumor *BRAF* mutations (43.6% vs. 53.1%, *p* = 0.361) or BRAF(±MEK) inhibitors received in previous or later treatment lines (Table S1).

**FIGURE 1:**
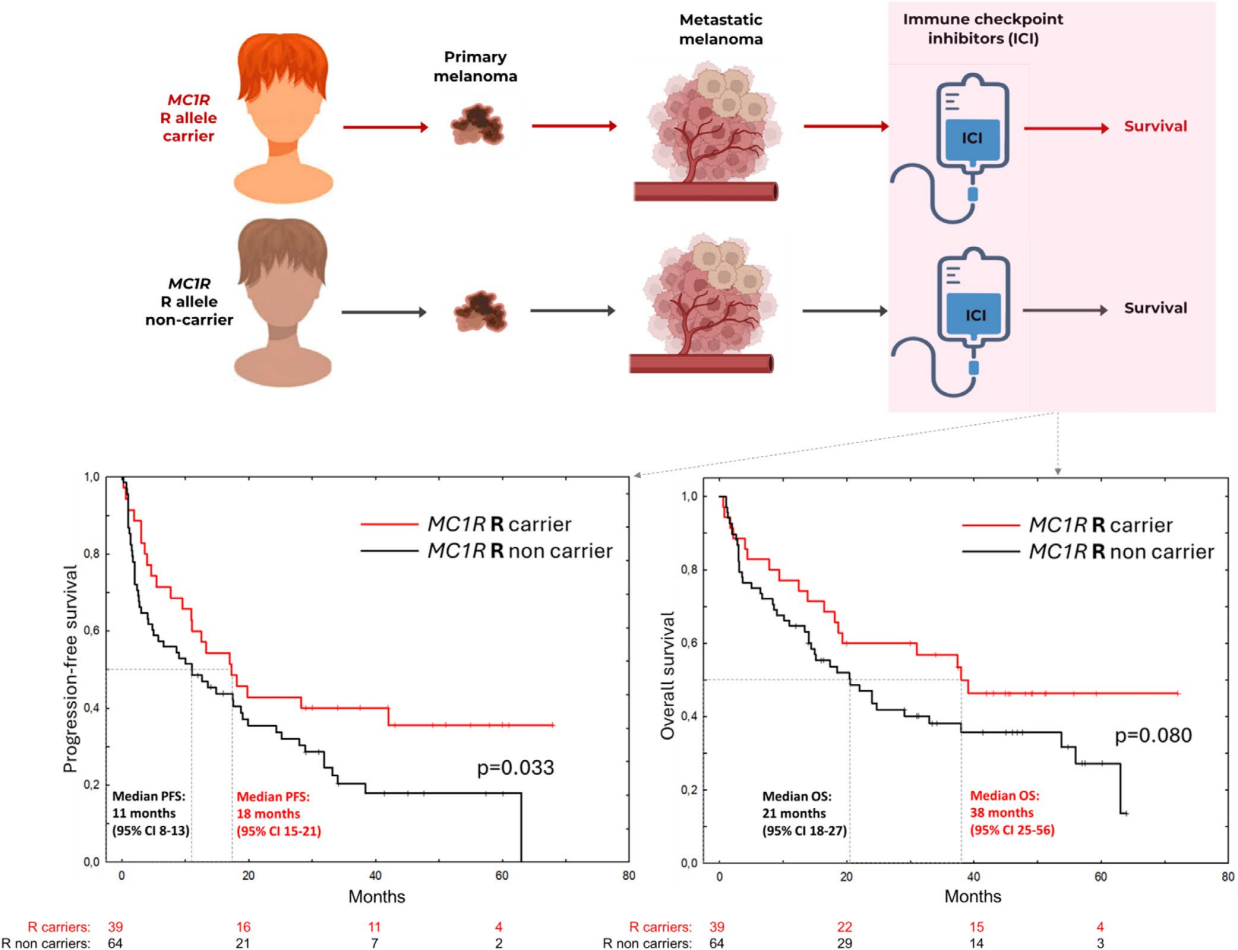
Study overview and Kaplan–Meier curves demonstrating the effect of *MC1R* R germline variants on progression‐free survival and overall survival in patients with metastatic melanoma treated with immune checkpoint inhibitors.

**TABLE 1:** Univariate and multivariate analysis on the effect of *MC1R* R germline variants on progression‐free survival and overall survival in ICI‐treated patients with metastatic melanoma by Cox proportional hazards regression model.

	Progression‐free survival				Overall survival			
	HR	95% CI		*p*	HR	95% CI		*p*
		Lower	Upper			Lower	Upper	
*MC1R*‐R‐carriers versus non carriers								
Unadjusted	0.60	0.37	0.98	0.043	0.63	0.37	1.08	0.091
Adjusted for demographic factors (age and sex)	0.60	0.37	0.99	0.044	0.62	0.36	1.06	0.081
Adjusted for phenotypic features (hair color and skin type)	0.56	0.31	1.03	0.064	0.55	0.28	1.08	0.084
Adjusted for primary melanoma characteristics (site, subtype, T stage and *BRAF* mutation)	1.05	0.52	2.10	0.891	0.93	0.44	1.96	0.849
Adjusted for tumor related prognostic factors at start of ICI (Stage and LDH)	0.66	0.41	1.09	0.102	0.74	0.43	1.27	0.275
Adjusted for treatments (previous treatments and ICI regime received)	0.56	0.34	0.92	0.022	0.61	0.35	1.04	0.067

Abbreviations: HR, hazard ratio; ICI, immune checkpoint inhibitors; LDH, lactate dehydrogenase in serum.

As individuals with more UV‐sensitive skin types are more prone to burn, their melanomas can accumulate more UV‐associated mutations and a broader repertoire of tumor antigens, and hence potentially increased sensitivity to ICI. TMB is a known positive predictive marker for ICI efficacy (Hugo et al. 2016; McGranahan et al. 2016; Snyder et al. 2014). Germline *MC1R* variants, in particular the *R* alleles, have also been associated with higher tumor mutational burden (TMB) in primary melanoma tumors (Johansson et al. 2017; Robles‐Espinoza et al. 2016). Interestingly, this association was seen for both UVR‐signature changes and non‐UVR base‐pair substitutions. The effect from *MC1R R* alleles on ICI response and survival is hence probably not only mediated through pathways involved in pigmentation and melanin synthesis but also by DNA damage repair, that affect the pathogenesis of the melanoma resulting in different histologic subtypes and varying tumor mutation burden. Since the tumor PD‐L1 status is not considered essential for granting ICI treatment in melanoma, this test hasn't been routine in Sweden and wasn't available in our cohort. It would, however, be informative to study if *MC1R* status correlates with expression of PD‐L1 or other immune checkpoints in the tumor microenvironment that could affect the tumor immunogenicity.

To summarize, ICI treated *MC1R*‐R‐carriers in this study had a favorable survival compared to non‐carriers. To our knowledge, this study is the first to address the efficacy of ICI in relation to *MC1R* R allele status. A limitation of this study is the relatively small number of patients, and larger‐scale studies from different populations on germline *MC1R* variants as a predictive marker for ICIs in metastatic melanoma are warranted. It would further be of interest to study if the germline *MC1R* status can predict the efficacy of different ICI regimens (Larkin et al. 2015; Tawbi et al. 2022), for example, if PD‐1 inhibitor monotherapy would be sufficient in R‐carriers, whilst combination therapies with PD‐1 inhibitors and CTLA‐4 or LAG‐3 would be required in non‐carriers.

## Author Contributions

Conceptualization: H.H., S.E.B., F.C.S., V.H. Data curation: H.H., V.H. Formal analysis: H.H., V.H., M.Y. Funding acquisition: H.H., V.H. Investigation: All authors. Methodology: H.H., V.H., S.E.B., F.C.S., V.G. Project administration: H.H., V.H., S.E.B., F.C.S., M.Y. Software: H.H., V.H. Resources: H.H., V.H., S.E.B., F.C.S., M.Y. Supervision: H.H., V.H. Validation: H.H., V.H., M.Y. Visualization: H.H. Writing – original draft: H.H. Writing – review and editing: All authors.

## Ethics Statement

The study was conducted in accordance with Good Clinical Practice with informed consent from all patients and was approved by the Stockholm Regional Ethics Committee.

## Conflicts of Interest

The authors declare no conflicts of interest.

## Supporting information


**Data S1:** pcmr70050‐sup‐0001‐DataS1.docx.

## Data Availability

The data that support the findings of this study are available from the corresponding author, upon reasonable request.
