# Supplementary material for: Germline MC1R Variant Status and Efficacy of Immune Checkpoint Inhibitors in Patients With Advanced Melanoma
Source: Pigment Cell Melanoma Res. 2025 Sep 9;38(5):e70050. doi: 10.1111/pcmr.70050 (PMC12420945; doi:10.1111/pcmr.70050)
Supplement: Supplementary file 1 — Data S1: pcmr70050‐sup‐0001‐DataS1.docx. [file PCMR-38-0-s001.docx]

Muyi Yang, Suzanne Egyhazi Brage, Jan Lapins, Vitali Grozman, Fernanda Costa Svedman, Veronica Höiom, Hildur Helgadottir

*Germline MC1R variant status and efficacy of immune checkpoint inhibitors*

*in patients with advanced melanoma*

**Supplementary file:**

**Supplementary Methods**

**Supplementary Results**

**References**

**Supplementary Table S1**

**Supplementary Table S2**

**Supplementary Table S3**

**SUPPLEMENTARY METHODS**

*Patient cohort*

Blood samples were collected from patients with unresectable metastatic cutaneous melanoma, treated with ICI (anti-CTLA-4 and/or anti-PD-1) at the Department of Oncology, Karolinska University Hospital, Stockholm, Sweden in the years 2012-2021. Collected data included patient characteristics (age, sex, hair color, skin type by Fitzpatrick classiﬁcation and family or personal history of melanoma), characteristics of the primary melanoma (body site, melanoma histopathological subtype and classification of the primary melanoma according to American Joint Committee on Cancer (AJCC), Eighth Edition) and characteristics at start of ICI treatment for metastatic melanoma (tumor stage (AJCC, Eighth Edition), plasma lactate dehydrogenase (LDH) value, tumor *BRAF* mutation status, previous lines of treatments and ICI regime chosen for the patient (Fitzpatrick, 1988; Gershenwald et al., 2017).

*DNA preparation and Sanger sequencing*

Genomic DNA was prepared, and Sanger sequencing was performed as previously described (Hoiom et al., 2009). Briefly, 1-2 ml of peripheral blood sample per patient was used, according to manufacturer’s protocol (QIAamp^®^ DNA Mini Kit, Qiagen). Each polymerase chain reaction was performed using 100 ng of genomic DNA in a 20 µL system, according to protocol from Phusion High-Fidelity DNA Polymerase, Thermofisher Scientific. PCR products were enzymatically cleaned up using a combination of Exonuclease I (20 U/µL) and FastAP Thermosensitive Alkaline Phosphatase (1U/µL), before being subject to sequencing reactions (BigDye^™^ Terminator v3.1 Cycle Sequencing Kit, Thermofisher Scientific). Bidirectional sanger sequencing was performed on ABI 3730 PRISM^®^ DNA Analyzer at KIGene Core Facility at Karolinska Institutet. Two pairs of genomic primers (upstream and downstream) were designed to sequence *MC1R* gene. Genomic DNA primers used in this study are as follows: upstream forward: 5’-CCTGGCAGCACCATGAACTA-3’, reverse: 5’-AGTAGGCGATGAAGAGCGTG-3’; downstream forward: 5’-CTTCTACGCACTGCGCTACC-3’, reverse: 5’-GTCCGCGCTTCAACACTTTC-3’. Sanger sequencing data was analyzed using Mutation Surveyor V5.1.2. Classification of *MC1R* *R* alleles was done accordingly to the paper by Robles et.al where *MC1R* variants Asp84Glu, Asp121Glu, Arg151Cys, Arg151Gly, Ile155Thr, Arg160W, Asp294His were classified as *R* alleles (Robles-Espinoza et al., 2016).

*Follow-up and statistical analyses*

Patients with metastatic cutaneous melanoma included in the study were treated with ICIs and followed-up according to clinical routine at the Department of Oncology at the Karolinska University Hospital. The routine follow-up included monthly clinical assessments and radiological evaluations every third month. For this study, patients were grouped based on *MC1R* R allele status and followed groupwise until the year 2024 for treatment response, progression-free survival (PFS) and overall survival (OS). Best response to treatment was based on radiological investigations (CT, MRI and/or positron emission (PET) CT tomography) evaluated by a radiologist. The evaluation of treatment response was carried out according to Response Evaluation Criteria in Solid Tumors (RECIST) 1.1 criteria (Eisenhauer et al., 2009). Response rate (RR) was defined as the frequency of patients with partial (PR) or complete responses (CR). Disease control rate was defined as the frequency of patients with PR, CR or stable disease (SD). PFS was defined as the time from treatment start until the date of confirmed progression or date of death or last follow-up. OS was defined as the time from treatment start until date of death or last follow-up. The patients were grouped for the presence of germline *MC1R* *R* variants (≥1 or 0). Patient and tumor characteristics and treatment responses in *MC1R*-R-carriers and non-carriers were compared with Chi-square test for categorical variables and Student's t-test for continuous variables. For survival plots, Kaplan-Meier analyses with Log-rank P values were computed. Cox proportional hazards regression models, unadjusted and adjusted were used to assess survival in the study cohorts. Hazard ratios (HR) and 95% confidence intervals (CI) were calculated. All statistical tests were two-sided. P values under 0.05 were considered statistically significant. Statistical analyses were performed with StatSoft Statistica software, version 13.

**SUPPLEMETARY RESULTS**

*MC1R variants*

The identified *MC1R* variant were Asp84Glu (n=2), Asp121Glu (n=2), Arg151Cys (n=18), Arg151Gly (n=1), Ile155Thr (n=4), Arg160W (n=15) and Asp294His (n=3), where six patients had two different *MC1R* R variants.

**References**

Eisenhauer, E. A., Therasse, P., Bogaerts, J., Schwartz, L. H., Sargent, D., Ford, R., Dancey, J., Arbuck, S., Gwyther, S., Mooney, M., et al. (2009). New response evaluation criteria in solid tumours: revised RECIST guideline (version 1.1). Eur J Cancer *45***,** 228-47.

Fitzpatrick, T. B. (1988). The validity and practicality of sun-reactive skin types I through VI. Arch Dermatol *124***,** 869-71.

Gershenwald, J. E., Scolyer, R. A., Hess, K. R., Sondak, V. K., Long, G. V., Ross, M. I., Lazar, A. J., Faries, M. B., Kirkwood, J. M., Mcarthur, G. A., et al. (2017). Melanoma staging: Evidence-based changes in the American Joint Committee on Cancer eighth edition cancer staging manual. CA Cancer J Clin *67***,** 472-492.

Hoiom, V., Tuominen, R., Kaller, M., Linden, D., Ahmadian, A., Mansson-Brahme, E., Egyhazi, S., Sjoberg, K., Lundeberg, J., and Hansson, J. (2009). MC1R variation and melanoma risk in the Swedish population in relation to clinical and pathological parameters. Pigment Cell Melanoma Res *22***,** 196-204.

Robles-Espinoza, C. D., Roberts, N. D., Chen, S., Leacy, F. P., Alexandrov, L. B., Pornputtapong, N., Halaban, R., Krauthammer, M., Cui, R., Timothy Bishop, D., et al. (2016). Germline MC1R status influences somatic mutation burden in melanoma. Nat Commun *7***,** 12064.

| **Supplementary Table S1. Characteristics of patients with metastatic melanoma depending on their germline *MC1R* genotype** | | | | | | | | | | |
| --- | --- | --- | --- | --- | --- | --- | --- | --- | --- | --- |
|  |  |  | *MC1R*-R-carriers | | |  | *MC1R*-R-non-carriers | | |  |
|  |  |  |  |  |  |  |  |  |  | *P* Value |
| **PATIENT CHARACTERISTICS IN RELATION TO THE PRIMARY MELANOMA** | | | |  |  |  |  |  |  |  |
|  | Sex, n (%) | |  |  |  |  |  |  |  |  |
|  |  | Female | 15 |  | 38.5% |  | 25 |  | 39.1% | 0.952 |
|  |  | Male | 24 |  | 61.5% |  | 39 |  | 60.9% |  |
|  | Age at melanoma diagnosis, median (range) | |  |  |  |  |  |  |  |  |
|  |  | Age, years (range) | 64 (36-83) | | |  | 64 (28-84) | | | 0.555 |
|  | Fitzpatrick phototype, n (%) | |  |  |  |  |  |  |  |  |
|  |  | I-II | 22 |  | 68.8% |  | 20 |  | 42.6% | 0.020 |
|  |  | III-IV | 10 |  | 31.3% |  | 27 |  | 57.4% |  |
|  |  | Missing | 7 |  |  |  | 17 |  |  |  |
|  | Hair color, n (%) | |  |  |  |  |  |  |  |  |
|  |  | Red | 6 |  | 20.0% |  | 2 |  | 4.5% | 0.044 |
|  |  | Blond | 16 |  | 53.3% |  | 28 |  | 63.6% |  |
|  |  | Brown or black | 8 |  | 26.7% |  | 14 |  | 31.8% |  |
|  |  | Missing | 9 |  |  |  | 20 |  |  |  |
|  | Family or personal history of previous melanoma, n (%) | |  |  |  |  |  |  |  |  |
|  |  | Yes | 8 |  | 24.2% |  | 9 |  | 17.0% | 0.410 |
|  |  | No | 25 |  | 75.8% |  | 44 |  | 83.0% |  |
|  |  | Missing | 6 |  |  |  | 11 |  |  |  |
| **PRIMARY TUMOR CHARACHTERISTICS** | | |  |  |  |  |  |  |  |  |
|  | Primary tumor known or unknown, n (%) | |  |  |  |  |  |  |  |  |
|  |  | Primary tumor known | 35 |  | 89.7% |  | 47 |  | 73.4% | 0.030 |
|  |  | Primary tumor unknown* | 4 |  | 10.3% |  | 17 |  | 26.6% |  |
|  | Site of primary melanoma, n (%)** | |  |  |  |  |  |  |  |  |
|  |  | Lower extremity | 17 |  | 48.6% |  | 16 |  | 34.0% | 0.317 |
|  |  | Upper extremity | 5 |  | 14.3% |  | 4 |  | 8.5% |  |
|  |  | Head and neck | 3 |  | 8.6% |  | 8 |  | 17.0% |  |
|  |  | Trunk | 10 |  | 28.6% |  | 19 |  | 40.4% |  |
|  | Type of primary melanoma, n (%)** | |  |  |  |  |  |  |  |  |
|  |  | Superficial spreading melanoma (SSM) | 16 |  | 61.5% |  | 10 |  | 27.8% | 0.022 |
|  |  | Nodular melanoma (NM) | 8 |  | 30.8% |  | 17 |  | 47.2% |  |
|  |  | Acral lentiginous melanoma (ALM) | 1 |  | 3.8% |  | 3 |  | 8.3% |  |
|  |  | Lentigo maligna melanoma (LMM) | 0 |  | 0.0% |  | 4 |  | 11.1% |  |
|  |  | Non-classifiable | 1 |  | 3.8% |  | 2 |  | 5.6% |  |
|  |  | Missing | 9 |  |  |  | 12 |  |  |  |
|  | Classification of the primary melanoma, n (%)** | |  |  |  |  |  |  |  |  |
|  |  | T1-T2 | 12 |  | 37.5% |  | 15 |  | 37.5% | 1.000 |
|  |  | T3-T4 | 20 |  | 62.5% |  | 25 |  | 62.5% |  |
|  |  | Missing | 7 |  |  |  | 7 |  |  |  |
|  | *BRAF* V600 mutation in tumor, n (%) | |  |  |  |  |  |  |  |  |
|  |  | Yes | 17 |  | 43.6% |  | 34 |  | 53.1% | 0.361 |
|  |  | No | 22 |  | 56.4% |  | 30 |  | 46.9% |  |
| **Supplementary Table S1. (continued)**  **PATIENT CHARACTERISTICS IN RELATION TO ICI TREATMENT** | | |  |  |  |  |  |  |  |  |
|  | Age at start of immunotherapy for metastatic melanoma | |  |  |  |  |  |  |  |  |
|  |  | Age, years (range) | 71 (47-84) | | |  | 70 (29-84) | | | 0.343 |
|  | Tumor stage, n (%) | |  |  |  |  |  |  |  |  |
|  |  | M1a or M1b | 22 |  | 56.4% |  | 26 |  | 40.6% | 0.112 |
|  |  | M1c or M1d | 17 |  | 43.6% |  | 38 |  | 59.4% |  |
|  | LDH, n (%) | |  |  |  |  |  |  |  |  |
|  |  | Normal LDH | 19 |  | 48.7% |  | 27 |  | 42.2% | 0.513 |
|  |  | Elevated LDH | 20 |  | 51.3% |  | 37 |  | 57.8% |  |
|  | Previous lines of treatment, n (%) | |  |  |  |  |  |  |  |  |
|  |  | 0 previous lines | 33 |  | 84.6% |  | 49 |  | 76.6% | 0.306 |
|  |  | ≥1 previous lines | 6 |  | 15.4% |  | 15 |  | 23.4% |  |
|  | BRAF(±MEK) | | 4 |  |  |  | 11 |  |  |  |
|  | Ipilimumab | | 3 |  |  |  | 4 |  |  |  |
|  | Chemotherapy | | 1 |  |  |  | 3 |  |  |  |
|  | Immunotherapy regime, n (%) | |  |  |  |  |  |  |  |  |
|  |  | PD-1 inhibitor monotherapy | 36 |  | 92.3% |  | 53 |  | 82.8% | 0.297 |
|  |  | CTLA-4 inhibitor monotherapy | 0 |  | 0.0% |  | 2 |  | 3.1% |  |
|  |  | CTLA-4 and PD-1 combination | 3 |  | 7.7% |  | 9 |  | 14.1% |  |
|  | Post-progression treatments | |  |  |  |  |  |  |  |  |
|  |  | 0 post-progression treatments | 30 |  | 76.9% |  | 42 |  | 65.6% | 0.207 |
|  |  | ≥1 post-progression treatments | 9 |  | 23.1% |  | 22 |  | 34.4% |  |
|  |  | BRAF±MEK | 5 |  |  |  | 9 |  |  |  |
|  |  | Ipilimumab(±nivolumab) | 2 |  |  |  | 7 |  |  |  |
|  |  | Chemotherapy | 2 |  |  |  | 8 |  |  |  |
|  |  |  |  |  |  |  |  |  |  |  |
|  | *At diagnosis metastatic disease and no diagnosis of primary melanoma | | | | | | | | | |
|  | **The unknown primary melanoma tumors are not accounted for in the site, type or classification of the primary tumor.  ICI: Immune checkpoint inhibitors, LDH: Lactate dehydrogenase in serum | | | | | | | | | |

| **Supplementary Table S2. Univariate analysis on the effect of host and melanoma specific factors on progression-free survival and overall survival by Cox proportional hazards regression model** | | | | | | | | | | | |  |
| --- | --- | --- | --- | --- | --- | --- | --- | --- | --- | --- | --- | --- |
|  |  |  |  |  |  |  |  |  |  |  |  |  |
|  |  |  | Progression-free survival | | | |  | Overall survival | | | |  |
|  |  |  |  | 95% CI | |  |  |  | 95% CI | |  |  |
|  | | | HR | Lower | Upper | *P* value |  | HR | Lower | Upper | *P* value |  |
| **Patient specific factors** | | |  |  |  |  |  |  |  |  |  |  |
|  | Sex, n (%) | |  |  |  |  |  |  |  |  |  |  |
|  |  | Female vs. male | 1.09 | 0.68 | 1.74 | 0.712 |  | 1.44 | 0.87 | 2.38 | 0.153 |  |
|  | Age at start of immunotherapy | |  |  |  |  |  |  |  |  |  |  |
|  |  | ≤65 vs. >65 | 0.82 | 0.51 | 1.32 | 0.416 |  | 0.67 | 0.40 | 1.12 | 0.124 |  |
|  | Skin type | |  |  |  |  |  |  |  |  |  |  |
|  |  | I-II vs. III-IV | 0.99 | 0.58 | 1.67 | 0.963 |  | 0.79 | 0.45 | 1.40 | 0.423 |  |
|  | Hair color | |  |  |  |  |  |  |  |  |  |  |
|  |  | Red or Blond vs. Brown or black | 1.04 | 0.58 | 1.87 | 0.898 |  | 0.80 | 0.43 | 1.51 | 0.497 |  |
|  | Family or personal history of previous melanoma | |  |  |  |  |  |  |  |  |  |  |
|  |  | Yes vs. no | 0.92 | 0.49 | 1.73 | 0.785 |  | 0.97 | 0.49 | 1.89 | 0.919 |  |
|  |  |  |  |  |  |  |  |  |  |  |  |  |
|  |  |  |  |  |  |  |  |  |  |  |  |  |
| **Primary melanoma** | | |  |  |  |  |  |  |  |  |  |  |
|  | Primary tumor known | |  |  |  |  |  |  |  |  |  |  |
|  |  | Primary tumor known vs. unknown | 0.67 | 0.39 | 1.15 | 0.144 |  | 1.00 | 0.53 | 1.88 | 0.998 |  |
|  | T stage of primary melanoma | |  |  |  |  |  |  |  |  |  |  |
|  |  | T1-T2 vs. T3-T4 | 1.43 | 0.80 | 2.57 | 0.231 |  | 1.70 | 0.92 | 3.12 | 0.089 |  |
|  | Site of primary melanoma | |  |  |  |  |  |  |  |  |  |  |
|  |  | Lower extremity vs. other sites | 0.98 | 0.57 | 1.67 | 0.929 |  | 1.09 | 0.62 | 1.91 | 0.756 |  |
|  |  | Upper extremity vs. other sites | 0.75 | 0.32 | 1.76 | 0.511 |  | 0.87 | 0.37 | 2.05 | 0.752 |  |
|  |  | Head and neck vs. other sites | 1.35 | 0.64 | 2.87 | 0.433 |  | 1.30 | 0.58 | 2.89 | 0.528 |  |
|  |  | Trunk vs. other sites | 1.02 | 0.58 | 1.77 | 0.957 |  | 0.85 | 0.46 | 1.57 | 0.602 |  |
|  | Type of primary melanoma | |  |  |  |  |  |  |  |  |  |  |
|  |  | SSM vs. NM | 0.92 | 0.46 | 1.85 | 0.824 |  | 0.78 | 0.37 | 1.63 | 0.508 |  |
|  |  | SSM vs. other subtypes | 0.74 | 0.39 | 1.37 | 0.334 |  | 0.70 | 0.36 | 1.36 | 0.293 |  |
|  |  | SSM or NM vs. other subtypes | 0.48 | 0.24 | 0.98 | 0.043 |  | 0.56 | 0.26 | 1.24 | 0.154 |  |
|  | BRAF mutation in tumor, n (%) | |  |  |  |  |  |  |  |  |  |  |
|  |  | Yes vs. no | 0.77 | 0.49 | 1.23 | 0.275 |  | 0.78 | 0.47 | 1.29 | 0.334 |  |
| **Supplementary Table S2 (continued)**  **Metastatic melanoma at start of immunotherapy** | | |  |  |  |  |  |  |  |  |  |  |
|  | Tumor stage, n (%) | |  |  |  |  |  |  |  |  |  |  |
|  |  | M1a-M1b vs. M1c-M1d | 0.42 | 0.26 | 0.68 | <0.001 |  | 0.41 | 0.24 | 0.70 | 0.001 |  |
|  | LDH, n (%) | |  |  |  |  |  |  |  |  |  |  |
|  |  | Normal vs. elevated LDH | 0.49 | 0.30 | 0.79 | 0.003 |  | 0.47 | 0.28 | 0.79 | 0.005 |  |
|  | Previous lines of treatment, n (%) | |  |  |  |  |  |  |  |  |  |  |
|  |  | 0 vs. ≥1 previous lines | 0.55 | 0.32 | 0.94 | 0.029 |  | 0.55 | 0.31 | 0.98 | 0.043 |  |
|  | Immunotherapy regime received | |  |  |  |  |  |  |  |  |  |  |
|  |  | PD-1 or CTLA-4 monotherapy vs. PD-1/CTLA-4 combination | 1.25 | 0.57 | 2.73 | 0.578 |  | 0.90 | 0.41 | 1.99 | 0.799 |  |
| HR: Hazard ratio SSM: Superficial spreading melanoma, NM: Nodular melanoma, LDH: Lactate dehydrogenase in serum | | | | | | | | | | | |  |

| **Supplementary Table S3. Responses to immune checkpoint inhibitor therapy in patients with metastatic melanoma depending on their germline *MC1R* genotype** | | | | | | | | | |
| --- | --- | --- | --- | --- | --- | --- | --- | --- | --- |
|  |  | *MC1R*-R-carriers | | |  | *MC1R*-R-non-carriers | | |  |
|  |  |  |  |  |  |  |  |  | *P* value |
| Best overall response, n (%) | |  |  |  |  |  |  |  |  |
|  | Complete response (CR) | 9 |  | 23.1% |  | 12 |  | 18.8% |  |
|  | Partial response (PR) | 12 |  | 30.8% |  | 20 |  | 31.3% |  |
|  | Stable disease (SD) | 8 |  | 20.5% |  | 6 |  | 9.4% |  |
|  | Progressive disease (PD) | 10 |  | 25.6% |  | 26 |  | 40.6% |  |
|  |  |  |  |  |  |  |  |  |  |
| Response rate (CR+PR), % | | 21 |  | 53.8% |  | 32 |  | 50.0% | 0.702 |
| Disease control rate (CR+PR+SD), % | | 29 |  | 74.4% |  | 38 |  | 59.4% | 0.056 |
